# Efficacy and Safety of the Combination of Pravastatin and Sorafenib for the Treatment of Advanced Hepatocellular Carcinoma (ESTAHEP Clinical Trial)

**DOI:** 10.3390/cancers12071900

**Published:** 2020-07-14

**Authors:** Ioana Riaño, Leticia Martín, Maria Varela, Trinidad Serrano, Oscar Núñez, Beatriz Mínguez, Pedro M. Rodrigues, Maria J. Perugorria, Jesus M. Banales, Juan I. Arenas

**Affiliations:** 1 Department of Liver and Gastrointestinal Diseases, Clinical Research Unit, Donostia University Hospital-Biodonostia Health Research Institute, 20014 San Sebastian, Spain; IOANA.RIANOFERNANDEZ@osakidetza.eus (I.R.); leticia.martin@asef.es (L.M.); pedro.rodrigues@biodonostia.org (P.M.R.); matxus.perugorria@biodonostia.org (M.J.P.); 2 Digestive Service, Hepatology Unit, Asturias Central University Hospital, The University Institute of Oncology of Asturias (IUOPA), FINBA, 33006 Oviedo, Spain; maria.varela.calvo@gmail.com; 3 Liver Unit, Lozano Blesa University Hospital-Aragon Health Research Institute, 50009 Zaragoza, Spain; utra.hcu@salud.aragon.es; 4 Digestive Service, Infanta Sofía University Hospital, 28703 San Sebastián de los Reyes-Madrid, Spain; onumar@gmail.com; 5 Liver Unit, Department of Medicine, Hospital Universitari Vall d’Hebron, Vall d´Hebron Institut of Research (VHIR), Universitat Autònoma de Barcelona, 08035 Barcelona, Spain; bminguez@vhebron.net; 6 Carlos III Health Institute (ISCIII), Centro de Investigación Biomédica en Red de Enfermedades Hepáticas y Digestivas (CIBERehd), 28220 Madrid, Spain; 7 Department of Medicine, Faculty of Medicine and Nursing, University of the Basque Country, University of the Basque Country (UPV/EHU), 48940 Lejona, Spain; 8 Basque Foundation for Science, IKERBASQUE, 48013 Bilbao, Spain

**Keywords:** hepatocellular carcinoma, sorafenib, pravastatin, randomized clinical trial, overall survival, time to progression, prognostic factors

## Abstract

Pravastatin has demonstrated anti-tumor activity in preclinical and clinical studies. This multicentric randomized double-blind placebo-controlled phase II study (NCT01418729) investigated the efficacy and safety of sorafenib + pravastatin combination on the overall survival (OS) and time to progression (TTP) of patients with advanced hepatocellular carcinoma (aHCC). A total of 31 patients were randomized. Median OS did not differ between both groups (12.4 months for the sorafenib + pravastatin group vs. 11.6 months for the control group). Of note, however, the radiological TTP was higher in patients treated with sorafenib + pravastatin than in the control group (9.9 months vs. 3.2 months; *p* = 0.008). Considering all the study population, the presence of portal vein thrombosis (PVT) was associated with worse OS, being lower in patients with PVT compared to patients without PVT (6.3 months vs. 14.8 months; *p* = 0.026). Data also showed a decrease in OS in patients with vascular invasion (VI) compared to patients who did not present it (6.3 months vs. 14.8 months; *p* = 0.041). The group of patients without dermatological events (DE) showed lower OS (6.9 months vs. 14.5 months; *p* = 0.049). In conclusion, combination of sorafenib + pravastatin was safe and well-tolerated, prolonging the TTP of patients with aHCC but not improving the OS compared to sorafenib + placebo. The absence of PVT and VI and the development of DE are positive prognostic factors of sorafenib response.

## 1. Introduction

Hepatocellular carcinoma (HCC) is the most common liver cancer worldwide [1,2], the sixth most common neoplasm, and the third main cause of cancer-related death [3]. The incidence of HCC has been rising globally over the last 20 years and is expected to increase in the future; thus, the World Health Organization estimates that more than 1 million patients will die from liver cancer in 2030 [4,5]. The majority of HCCs develop in patients with underlying chronic liver disease and the main risk factors are the presence of cirrhosis, hepatitis B or C virus (HBV or HCV) infection, chronic alcohol abuse, metabolic syndrome (obesity, type 2 diabetes mellitus), non-alcoholic fatty liver disease (NAFLD) and aflatoxin exposure [1,2]. Currently, although the control of viral agents is improving, the prevalence of lifestyle risk factors is increasing [1]. 

The best durable curative therapeutic option for patients with HCC is surgery (liver resection and transplantation) [6]. However, most patients are commonly diagnosed with unresectable HCC due to advanced-stage disease, high-risk comorbidities, or resource limitations [7]. For these patients, systemic therapy is indicated [7], and sorafenib has been the standard of care in first-line treatment and is currently widely used for the treatment of patients with advanced HCC (aHCC) [8].

Sorafenib is an oral tyrosine kinase inhibitor (TKI) that targets kinases involved in angiogenesis and tumor proliferation pathways implicated in the molecular pathogenesis of HCC (i.e., Raf-1, B-Raf, vascular endothelial growth factor receptor 1–3, and platelet-derived growth factor receptor β) [9]. Sorafenib has demonstrated its efficacy in prolonging the survival of patients with aHCC. Two major randomized phase III trials—one of them a multicenter clinical trial predominantly performed in Europe and the USA, and another conducted in the Asia-Pacific area—showed that sorafenib significantly increased overall survival (OS) and time to progression (TTP) compared to placebo [10,11]. Those results allowed for the approval of sorafenib as the standard treatment for patients with aHCC. More recently, the TKI lenvatinib was reported to be non-inferior to sorafenib in terms of OS benefit in this clinical setting [3]. However, the median OS remains poor and limited in both therapeutic settings [3]. Since most patients have unresectable disease, and given the clinical limitations of the available drugs, there is an urgent need for more effective systemic therapies [7]. In this regard, combination strategies involving sorafenib and other drugs [12] might constitute a promising approach and is currently getting attention in the field.

Statins are inhibitors of 3-hydroxy-3-methyl-glutaryl-coenzyme A (HMG-CoA) reductase that catalyzes the key limiting step in cholesterol biosynthesis. Inhibition of this enzyme blocks the production of mevalonate and its downstream metabolites. The mevalonate pathway is an important metabolic pathway that uses acetyl-CoA to produce sterols and isoprenoids that are essential for tumor growth and progression [13]. Oral and chronic administration of statins is approved and considered to be safe and effective for patients with hypercholesterolemia. Multiple studies have found an inverse relationship between statin use and the risk of developing different types of cancer, including colon, breast, pancreas, and prostate cancer [14]. In the past few years, several observational studies have also shown the preventive and therapeutic benefits of statins for patients with HCC reporting a consistent reduced risk of decompensation and death in patients receiving statins [12,15]; furthermore, statin administration has been associated with a reduction in the risk of developing HCC compared to statin nonusers [16,17].

Statins may exert multiple pleiotropic effects on HCC, including anti-proliferative, anti-oxidant, anti-inflammatory, and anti-fibrotic effects [12]. In particular, pravastatin has shown to inhibit HCC growth in vitro and in vivo by promoting apoptosis of tumor cells [18,19]. Furthermore, pravastatin is the only statin investigated in published clinical trials assessing the potential benefits of statins on HCC. Clinically, administering pravastatin as an adjuvant therapy was reported to improve the survival of patients with HCC in three different studies—(1) an open-label trial including patients with aHCC (mostly Child-Pugh B) treated with transarterial chemoembolization (TACE) and 5-fluorouracil, being then randomized to pravastatin or no treatment [20]; (2) a randomized phase II trial in aHCC treated with octreotide followed by octreotide or pravastatin or gemcitabine (median OS was significantly longer in pravastatin vs. gemcitabine) [21]; and (3) a prospective cohort of patients with aHCC treated with chemoembolization and pravastatin, compared to chemoembolization alone [22]. Of note, the combination of pravastatin and sorafenib was more effective than sorafenib alone in experimental models of HCC, decreasing tumor cell proliferation in vitro and in vivo [18], thus pinpointing the potential of sorafenib + pravastatin for the adjuvant treatment of HCC. In this regard, the hepatic safety profile of pravastatin and the limited risks of drug interactions with sorafenib [23] make this combination even more attractive.

This Phase II multicenter, double-blind trial was performed to evaluate the efficacy (OS and TTP) and safety of sorafenib and pravastatin combination in patients with aHCC that are eligible to receive systemic treatment with sorafenib.

## 2. Results

### 2.1. Patient Characteristics

**Figure 1:**
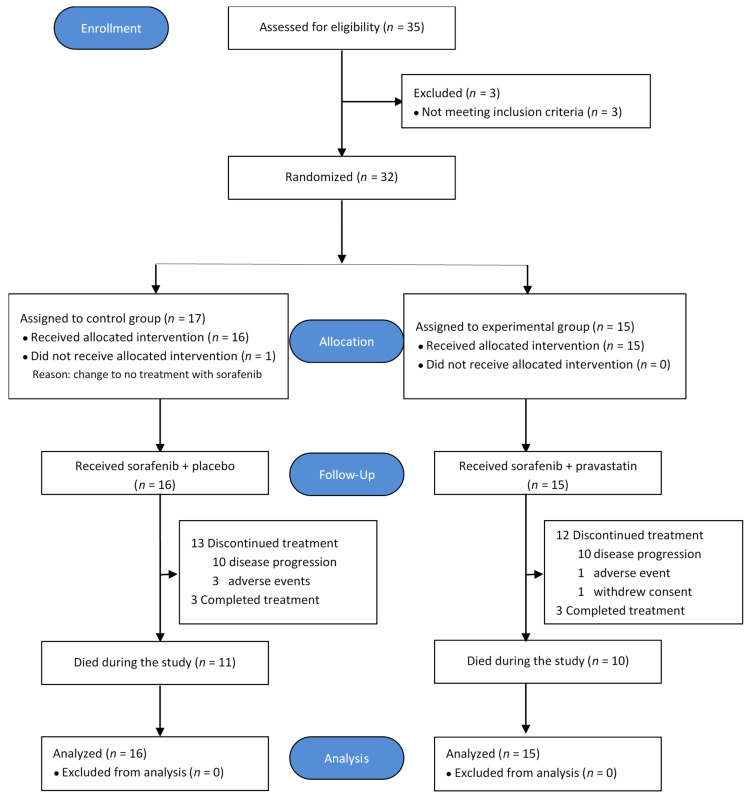
Flow chart of the ESTAHEP-2010 trial (CONSORT diagram).

From October 2011 to February 2016, 35 patients were screened, and 32 patients from five centers in Spain (Donostia University Hospital, Asturias Central University Hospital, Lozano Blesa University Hospital, Infanta Sofía University Hospital, Vall d´Hebron University Hospital) were randomized into control and experimental groups. A flow chart of the study population is shown in Figure 1. 

The mean age of the population was 61.4 years. Baseline characteristics were well balanced between treatment groups [i.e., Control (placebo and sorafenib) vs. Experimental (pravastatin and sorafenib)]. In the study population, 90% of the patients were Child A, 77% BCLC C, 42% presented with vascular invasion (VI), and 35% with portal vein thrombosis (PVT), in parallel with approximately 40% of the patients presenting extrahepatic metastases. Overall, 28 patients (90.3%) concomitantly displayed cirrhosis, mainly related to viral infection (61.3% of cases) and alcohol consumption (51.6% of cases). The main baseline characteristics are presented in Table 1.

### 2.2. Treatment

After randomization, 31 patients received at least one dose of the study treatment. The median and mean duration of the treatment in the sorafenib + placebo group were 102.5 days and 177.6 days, respectively. On the other hand, the median and mean treatment durations in the sorafenib + pravastatin group were 286 days and 251.2 days, respectively, while no statistically significant difference between both treatment groups (*p* = 0.254) was observed. Treatment was interrupted in 25 patients (80.6%) mainly due to disease progression. 

### 2.3. Efficacy

#### 2.3.1. Overall Survival

Considering the whole study population, 10 patients (32.3%) completed the study (five in each treatment group; 5/5), six of them completing all the treatment regimen (3/3), while 21 patients (67.7%) died before the last visit. Overall, mean OS was 11.5 months (344.9 days; 95% confidence interval [CI], 280.7–409.1), and the median OS was 12.4 months (373.0 days; 95% CI, 167.0–579.0), with an OS at 6, 12, and 18 months of 74.2%, 51.6%, and 32.3% respectively.

**Figure 2:**
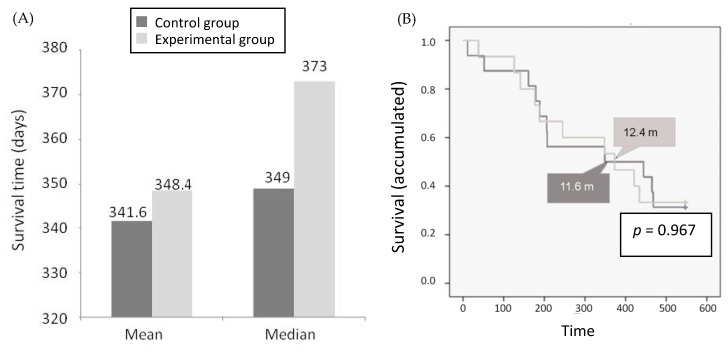
Survival by treatment group. (**A**) Survival (mean and median, days); (**B**) overall survival (OS) (median, days) estimated from the date of randomization to the date of death from any cause. Patients alive at the end of the study were censored at their last contact date.

The survival analysis is shown in Figure 2A. The mean survival of the control group was 11.4 months (341.6 days; 95% CI, 249.6–433.7 days) and 11.6 months (348.4 days; 95% CI, 259.3–437.5 days) for the experimental group. On the other hand, the median survival in the control group was of 11.6 months (349.0 days) and in the experimental group of 12.4 months (373.0 days). Overall, there was no significant difference in OS between the two experimental groups (*p* = 0.967) (Figure 2B).

**Figure 3:**
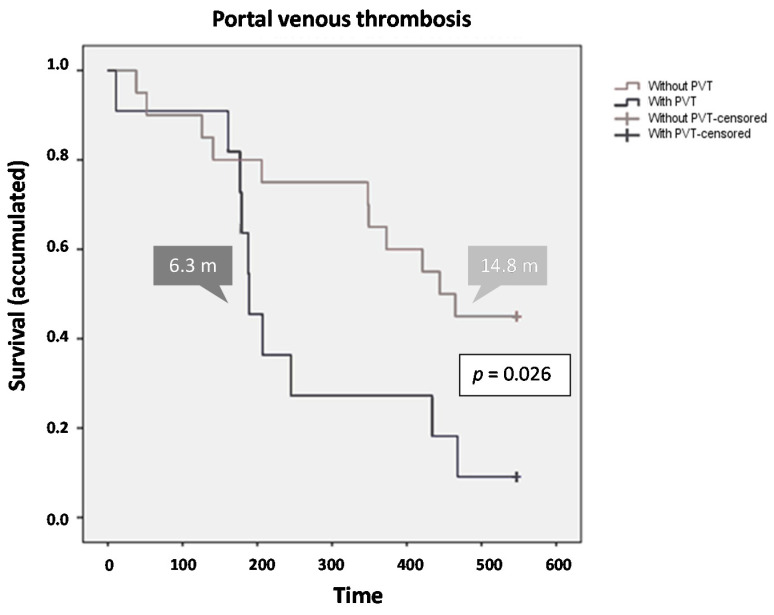
Survival by presence/absence of portal venous thrombosis.

Regarding the factors affecting survival, the presence of PVT and VI had a significant impact on the survival of the total population. A Cox regression analysis showed that PVT remained a strong negative prognostic factor for OS. Patients without PVT showed higher survival values [mean 13.1 months (394.3 days; 95% CI, 316.0–472.6 days), median 14.8 months (444.0 days; 95% CI 347.6–540.4 days)], with an end-of-study survival rate of 45%. In contrast, those patients with PVT [mean 8.5 months (255.1 days; 95% CI, 165.3–345.0 days), median 6.3 months (189.0 days; 95% CI, 158.8–219.2 days)], presented an end-of-study survival percentage of 9% (*p* = 0.026) (Figure 3). The assigned treatment did not influence in survival in respect to the presence/absence of PVT (*p* = 0.301).

**Figure 4:**
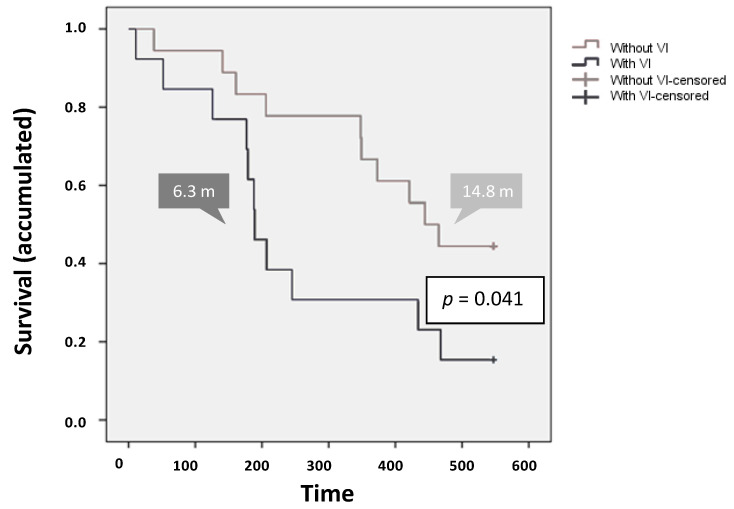
Survival by presence/absence of vascular invasion.

The impact of the presence of VI on survival rates was negative (*p* = 0.041). Specifically, in comparison with patients without VI, the presence of VI resulted in a decrease in the mean [13.6 months (406.8 ± 38.4 days; 95% CI, 331.5–482.0) vs. 8.6 months (259.2 ± 47.9 days; 95% CI, 165.3–353.2), respectively] and in the median [14.8 months (444 ± 46.7 days; 95% CI, 352.5–535.5) vs. 6.3 months (189 ± 16.8 days; 95% CI, 156.1–221.9), respectively] survival rates. Importantly, a 135% increase in survival was observed for patients without VI, compared to the population with VI (Figure 4).

#### 2.3.2. Tumor Response

**Figure 5:**
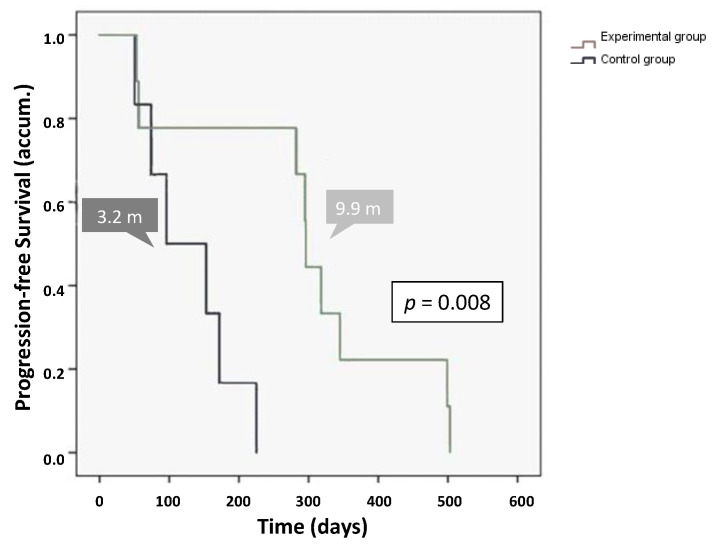
Radiological time to progression analysis by treatment group.

During the study, radiological progression was evident in 15 of the 31 patients. The mean and median radiological TTP in the total population were 7.6 months (227.8 ± 39.3 days; 95% CI, 150.7–304.9 days) and 7.5 months (225 ± 83.1 days; 95% CI, 62.2–387.8 days), respectively. At one-year after initiating the treatment, 58% of patients were free from disease progression. The median TTP was significantly longer in the experimental group [mean 9.8 months (294.1 ± 53.2 days) and median 9.9 months (296 ± 1.5 days)] compared to the control group [mean 4.3 months (128.3 ± 27.1 days) and median 3.2 months (96 ± 48.4 days)] (*p* = 0.008) (Figure 5).

During the study, symptomatic progression appeared in 21 of the 31 patients. In the whole study population, the mean of symptomatic TTP (TTSP) was 6.2 months (186.9 ± 30.5 days; 95% CI, 127.1–246.7 days), with a median of 4.6 months (137 ± 40.4 days; 95% CI, 57.75–216.25 days). The difference in the median TTSP was not statistically significant between the treatment and control groups (*p* = 0.393), presenting mean values of 154.1 and 223.0 days and median values of 111.0 and 137.0 days for the control and experimental groups, respectively.

### 2.4. Safety

During the study, 182 adverse events (AE) were reported, with 19 (10%) of them being considered serious adverse events (SAE). Importantly, their incidence was independent of the Child Pugh functional stage and BCLC tumor stage (Table 2). Ninety-five (52.2%) AEs were considered to be treatment-related. Among those, 92 (96.8%) were linked to sorafenib treatment, two (2.1%) to pravastatin, and one (1.1%) to the combination regimen. The most common treatment-related AEs were diarrhea, asthenia, anorexia/hyporexia, weight loss, hand-foot syndrome, rash, and itching.

Although the overall AE profiles of the two groups were similar, there were noticeable differences. In relation to the 19 SAEs, although not statistically significant, their incidence was higher in patients from the control group (63.16%) when compared to patients from the experimental group (36.84%). Four patients discontinued the treatment due to AEs: three of them were in the control group [asthenia (2), diarrhea and esophagitis (1)], and one was in the experimental group (anorexia and abdominal pain). All the registered deaths were due to disease progression.

Among the common AEs of special interest of any grade, the most relevant were dermatological events (DE, as pre-specified by protocol), with 51.6% of patients presenting dermatological toxicity (grades 1–3) at some point in the study. Episodes of hand-foot syndrome, skin rash, and pruritus occurred in a higher frequency, while alopecia, skin edemas and xerosis were less frequently observed. 

**Figure 6:**
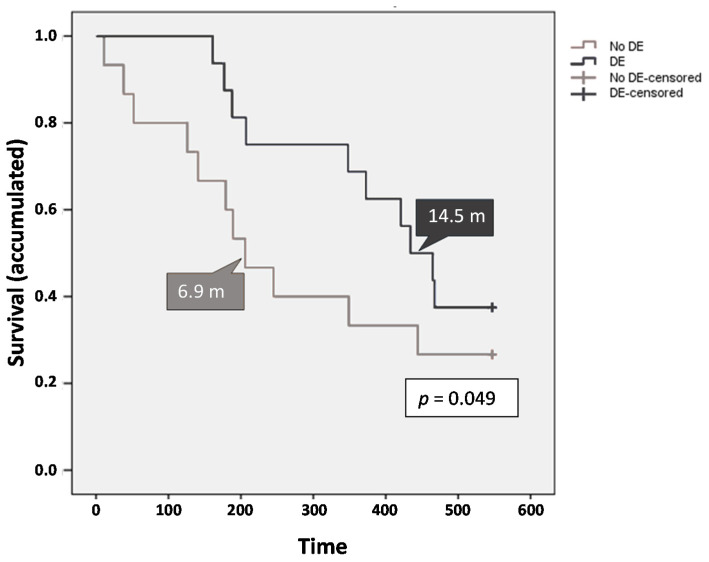
Overall survival according to dermatological toxicity.

The presence of DE within the 60 days of treatment was associated with an increased survival in the analysis. Patients were divided into two groups: patients who did not develop these adverse events and patients who experienced DE, showcasing statistically significant differences (*p* = 0.049). The group without dermatological toxicity exhibited lower OS [mean 9.3 months (277.9 days; 95% CI, 179.4–376.3 days); median 6.9 months (206.0 days; 95% CI, 122.7–289.3 days)], with a percentage of end-of-study survival rate of 27% compared to 38% in the group with dermatological toxicity (mean 13.6 months (407.7 days; 95% CI, 337.2–478.3 days); median 14.5 months (434.0 days; 95% CI, 347.8–520.2 days)]. The presence of DE was significantly associated with improved median OS, showing a 110% increase in the survival for patients with dermatological toxicity (Figure 6). This event was then confirmed as a prognostic factor. On the other hand, there was no significant difference in OS related to the presence of DE between the study groups.

A summary of survival results for the main variables are presented in the Table S1.

## 3. Discussion

The ESTAHEP study is the first double-blind, placebo-controlled, randomized trial reporting the safety and tolerability of the combination of pravastatin and sorafenib for the treatment of aHCC. Of note, this study showed a significant prolongation of TTP in the combination sorafenib + pravastatin when compared to sorafenib + placebo, but failed to demonstrate a benefit in OS. Moreover, this study showed that PVT and VI are associated with decreased OS and confirmed that the development of DE is a positive prognostic factor of sorafenib response.

In our Phase II study, the OS associated to sorafenib (mean 11.5 and median 12.4) was comparable to previously published Phase III clinical trials for aHCC [10,24], indicating that our study population behaves as expected when it comes to the response to sorafenib. However, similar to a recent Phase III clinical trial [23], the combination of sorafenib with pravastatin did not increase the OS of patients with aHCC, thus limiting its clinical impact. OS still represents the gold standard endpoint for trials in first-line treatment for aHCC; however, OS does not capture the full extent of anti-tumor effects. Increasing evidence points to the need to define other additional endpoints, including TTP, which may also impact on the disease evolution, as well as on the symptomatology and quality of life of patients. Moreover, results derived from these additional endpoints may have major impact in the design of further personalized treatment strategies based on different parameters (e.g., tumor stage, age and/or symptomatology), which could impact on the OS and quality of life of patients. Of note, in our study, the TTP was significantly higher in the experimental group (sorafenib + pravastatin) compared to the control group (placebo + sorafenib) (median 9.9 months vs. 3.2 months, respectively), which could be attributed to the previously reported anti-proliferative and pro-apoptotic properties of statins in HCC [12]. Indeed, simultaneous targeted inhibition of RAF/MEK/ERK with the combination of sorafenib and statins are known to induce potentiated effects in different tumor cell lines, inducing cell cycle arrest and apoptosis [25]. It is important to highlight that no differences regarding TTP were observed in previous studies with pravastatin and sorafenib [23,26]. The fact that in our study the combination of sorafenib + pravastatin increased the TTP in three months compared to a previous Phase III clinical trial including Child-Pugh A patients with advanced HCC [23] could be related to characteristics of the patients of our cohort, since our study population was five years younger (median age). In fact, higher age was previously associated with shorter TTP for regorafenib treatment in aHCC [27], although it should be noted that neither Child B patients nor those with extrahepatic metastases were included in this trial, as were included in our study. These data on TTP and OS, together with the evidence of multiple retrospective studies indicating the preventive effect of statins on cancer development and several clinical trials reporting positive results in OS with pravastatin treatment [20,21,22], prompt us to hypothesize that the therapeutic effects of statins could be more evident in preventing HCC development (i.e., in cirrhosis and/or after early HCC tumor resection) and/or treatment of earlier stages of carcinogenesis, rather than in the treatment of aHCC. In line with this, a randomized double-blinded, placebo-controlled Phase II trial will examine the effects of pravastatin use versus placebo after 12 months of treatment on HCC recurrence in patients with liver cirrhosis (NCT03219372). Notably, previous studies and preclinical evidences have assessed the potential beneficial anti-inflammatory and antifibrotic effects of statins as well as the rationale for the use of statins in chronic liver disease including the setting of liver cirrhosis [16,28]. In this regard, the therapeutic efficacy of pravastatin may delay HCC development and should be addressed in these settings in a near future, particularly in patients with early stage disease and/or after tumor resection, during which the effects would probably be even more noticeable. 

Moreover, clinical anticancer effect of statins would be more evident with higher doses, as suggested by several studies reporting that higher dosage and longer duration of statin use was associated with greater protective effects on the development of HCC [17,29,30]. Furthermore, dose-dependent effects of statins on angiogenesis were observed in murine models in vivo, presenting proangiogenic effects at low doses and antiangiogenic effects when used at high doses [31].

In addition, taking into consideration other previously reported positive results so far with pravastatin in HCC (i.e., in combination with TACE, octreotide or chemoembolization), novel combination treatments with statins warrant further exploration and validation of their potential effect halting the progression of HCC. 

The analysis of factors affecting survival indicated that the presence of PVT and VI had a significant impact in the survival on total study population. Thus, our study showed a decrease in OS associated to both parameters, which was independent of the assigned treatment. These data are in agreement with several retrospective studies that have reported this relationship [32,33] and with a recent study indicating that the presence of PVT is a predictive factor of poor survival in HCC [34]. 

On the other hand, the analysis of AEs has allowed us to affirm that the use of the combined therapy of pravastatin and sorafenib is safe and well-tolerated, as the registered AEs were mostly grade 1 and 2 according to the Common Terminology Criteria for Adverse Events (CTCAE), and only three episodes were observed as possibly related to pravastatin administration. Higher frequency of anorexia/hyporexia, weight loss, and rash were observed in the experimental group. However, fewer cases of ascites and gastrointestinal bleeding were noticed. Notably, the presence of lower cases of ascites within the pravastatin group potentially suggests the protective effect of pravastatin in the maintenance of Child–Pugh A status, which might contribute to maintain the disease well-compensated. In addition, there were no serious AEs related to the treatment under study, and the incidence of AEs recorded in the total population was higher in the control group (63.2%) than in the experimental group (36.8%). These data suggest that patients with aHCC do not have increased susceptibility to hepatotoxicity from statins and these results are consistent with a recent clinical trial in patients with aHCC treated with pravastatin 40 mg [23]. Further, recent observations have shown that patients with liver disease do not have a higher risk of statin-induced liver toxicity when compared to the general population [16,28]. The fact that the combination of sorafenib + pravastatin is safe and displays low toxicity is of pivotal importance in order to further deeply study its potential benefits in patients with HCC (i.e., earlier vs. advanced stages) in the future.

Among other relevant results obtained in this study, it is important to highlight that there was a significant positive correlation between the presence of DE and OS (median 14.5 months with DE vs. 6.9 months without DE); moreover, ~90% of these DE occurred within the first 60 days of sorafenib treatment. Indeed, our results are in accordance with previous studies showing similar results [35,36]. In addition, recent studies have reported a positive correlation between the achievement of a better radiological response to sorafenib treatment with the presence of early dermatologic reactions in HCC [37]. In line with this, our results regarding DE and OS validate, in the context of our clinical trial, previously published findings. The role of the DE is even more relevant if we consider the absence of correlation between TTP and OS in the ESTAHEP trial, which was previously shown in the SHARP and Asian-Pacific trials. In fact, the association between OS and DE has been reported in recent publications [35,36], showing that early incidence of DE in patients treated with sorafenib is a predictor of better OS. In this regard, as suggested by our results, the impact of DE is maintained regardless of the radiological tumor progression and can be linked with OS. 

Overall, the main limitation of our study was the sample size, mainly attributable to difficulties in the inclusion of patients, lowering the statistical power for multiple comparisons (e.g., TTSP). However, despite this, some of our results are in line with previously reported results including higher sample sizes, demonstrating that both the methodology used and the analyses performed in this study have been carried out in an adequate methodological and precise manner.

## 4. Materials and Methods

### 4.1. Study Population

This was a 12-center, randomized, double-blind, placebo-controlled parallel groups, phase II study. Inclusion criteria: adult patients with histologically, cytologically or radiologically confirmed HCC who had not received previously treatment with sorafenib and had to be candidates for systemic treatment; Eastern Cooperative Oncology Group (ECOG) performance status score ≤2; a Child–Pugh score of A or B7; life expectancy ≥12 weeks; and with an adequate and stable renal function (serum creatinine ≤1.5 × upper limit of normality-ULN). Exclusion criteria: patients who routinely (≥3 days/week) took some type of statin; statin hypersensitivity or contraindication; diagnosis within the previous five years with another type of tumor, except skin cancer other than melanoma or carcinoma in situ of the cervix or bladder; prior chemotherapy or radiotherapy for other tumors; underwent liver transplantation; participation in another clinical trial with any investigational agents within 6 months prior to study screening; pregnancy or breastfeeding; grade ≥2 peripheral neuropathy; gastroduodenal ulcer perforation or bleeding in the last month; uncontrolled intercurrent illness including, but not limited to, asthma, heart failure > grade IINYHA, uncontrolled arterial hypertension, uncontrolled arrhythmias or acute myocardial infarction in the previous six months; major hemorrhagic diseases; or psychiatric or physical illness/social situations that would limit compliance with study requirements. 

### 4.2. Trial Design and Treatment Allocation

Eligible patients were randomized in 1:1 ratio and stratified according to the center and risk factors (vascular invasion and extrahepatic metastases) to be treated as follows: experimental group (sorafenib 400 mg every 12 h and pravastatin 40 mg every day) and control group (sorafenib 400 mg every 12 h and placebo); each of the medicines used at the authorized doses. In both groups, treatment was initiated in the day of baseline visit. Patients received the treatment until death or treatment discontinuation due to SAE development, patient refusal or clinical and/or radiological progression. The maximum treatment duration was 18 months. 

### 4.3. Procedures

An 18-months treatment period was planned, in which the visits at the outpatient clinic were scheduled every eight weeks from the baseline visit. Physical examinations, clinical and analytical evaluations were performed every visit including: disease history, cirrhosis etiology, physical exam, ECOG performance status, Child-Pugh score, electrocardiogram, hematology, biochemistry, coagulation, alpha-fetoprotein, lipid profile, liver serological profile, and concomitant medications. Tumors were assessed by computerized tomography (CT) or magnetic resonance imaging (MRI) at baseline every 16 weeks (two visits) during the 18 months of treatment and at the end of treatment visit or at the early withdrawal visit. All response assessments were done locally by investigators according to modified Response Evaluation Criteria in Solid Tumors (RECISTm). A follow-up visit was performed 30 days after the last dose treatment. Safety data were collected continuously. Local laboratory assessments were done every visit since the selection visit and were graded according to CTCAE (version 3.0).

### 4.4. Outcomes

The primary study endpoint was overall survival (OS), defined as the time from randomization to death from any cause. Patients remaining alive were censored at the time of the final visit (data cut-off) and patients who had been lost during the follow-up were censored in the analysis at the date of their last contact. A secondary endpoint was the time to progression (TTP), defined as the time from randomization to radiological progression, according to RECISTm criteria; deaths during follow-up without evidence of radiological progression were censored. Other secondary endpoints included time to symptomatic progression (TTSP), defined as the time from randomization to symptom progression measured by the ECOG scale, considering the progression an increase in the ECOG-PS of ≥1 point and/or the development of symptomatic disease from asymptomatic disease; objective response rate, safety and prognostic factors of treatment response. 

Safety endpoints included the incidence and severity of adverse events (AEs) and serious AEs (SAEs) and relationship to study drug. The severity of AEs was assessed using the National Cancer Institute CTCAE version 3. Safety assessments included recording of vital signs, hematological and biochemical laboratory testing and electrocardiography. Prognostic factors were evaluated from baseline and during the study according to presence/absence of the studied factor.

### 4.5. Statistical Analysis

Efficacy was analyzed in the intention to treat (ITT) population, defined as all randomly assigned patients who received at least 1 dose of study treatment. The trial was designed to detect an OS increase of 20% with a power of 80% and a two-sided type I error of 0.05. The estimated dropout rate was 10%. These hypotheses revealed 216 patients to be enrolled. Descriptive statistics were reported as mean, median, standard deviation, and range. Correlations were analyzed by Pearson’s χ^2^ test if both variables were categorical and box plot if one variable was categorical and the other was quantitative. Survival data were analyzed using Kaplan–Meier method, compared by log-rank test and adjusted with a Cox regression model. The existence of statistically significant differences in continuous variables for two categories was measured with Mann–Whitney *U* test, as well as with the Kruskal–Wallis method for more than two categories. Statistical analyses were carried out with SPSS v.23 (IBM Analytics, Armonk, NY, USA).

### 4.6. Ethical Consideration and Registration

The study was performed in accordance with the guidelines of Good Clinical Practice and the principles of the Declaration of Helsinki. The clinical trial was conducted in compliance with the regulation of two Royal Decrees: RD223/2004 and RD1090/2015, the latter in force since 13 January 2016. The protocol was reviewed and approved by the Ethics Committee/Institutional Review Board for this study (CEIC-E) and the Spanish Agency of Medicines and Medical Devices (AEMPS) (Protocol name: ESTAHEP-2010). All patients signed an informed consent form before the participation in the study. Safety of trial subjects was monitored by an independent data safety monitoring board. This trial is registered at https://www.clinicaltrialsregister.eu/, EudraCT Number: 2010-024421-21, and at https://www.clinicaltrials.gov/, identifier: NCT01418729).

## 5. Conclusions

The ESTAHEP study is the first double-blind, placebo-controlled, randomized trial that has reported the safety and tolerability of the combination of pravastatin and sorafenib for the treatment of advanced HCC. The combination treatment of sorafenib + pravastatin in patients with aHCC did not improve the OS compared to patients under the standard of care treatment with sorafenib. However, the fact that this combined treatment was safe and non-toxic, and further improved the TTP in these patients, supports the design of future clinical studies to further explore new potential therapeutic strategies with this combination to prevent HCC development and further progression, as well as to validate previous benefits attributed to the combination of statins at different therapeutic regimens in patients with HCC. Moreover, this study validates that the absence of PVT and VI, and the development of DE, are positive prognostic factors of response to sorafenib.

## Figures and Tables

**Table 1:** Baseline characteristics of the study population.

Characteristic	Control Group (Sorafenib + Placebo) (*n* = 16)	Experimental Group (Sorafenib + Pravastatin) (*n* = 15)	Total (*n* = 31)
Age (years)	59.94 ± 12.57	63.00 ± 10.39	61.42 ± 11.48
Sex (M/F)	14 (87.5%)/2 (12.5%)	15 (100%)/0 (0%)	29 (93.5%)/2 (6.5%)
Albumin (g/L)	37.32 ± 8.38	40.33 ± 4.59	38.83 ± 6.82
Prothrombin activity (INR)	1.24 ± 0.39	1.07 ± 0.10	1.16 ± 0.30
Platelets (10^3^/μL)	180.62 ± 81.38	127.53 ± 64.90	154.93 ± 77.49
Total bilirubin ≤1.2 mg/dL >1.2 mg/dL Missing	1.07 ± 0.59 11 (68.8%) 4 (25.0%) 1 (6.2%)	1.24 ± 0.70 8 (53.3%) 7 (46.7%) 0 (0.0%)	1.16 ± 0.64 19 (61.3%) 11 (35.5%) 1 (3.2%)
Serum AFP ≤100 IU/mL >100 IU/mL Missing	2710.18 ± 4093.59 6 (37.5%) 8 (50.0%) 2 (12.5%)	1315.08 ± 3584.00 10 (66.7%) 4 (26.7%) 1 (6.6%)	2012.63 ± 3841.57 16 (51.6%) 12 (38.7%) 3 (9.7%)
Child-Pugh classification: Stage A Stage B	13 (81.2%) 3 (18.8%)	15 (100.0%) 0 (0.0%)	28 (90.3%) 3 (9.7%)
ECOG-PS: Grade 0 Grade 1	12 (75.0%) 4 (25.0%)	12 (80.0%) 3 (20.0%)	24 (77.4%) 7 (22.6%)
Portal thrombosis (Yes/No)	6 (37.5%)/10 (62.5%)	5 (33.3%)/10 (66.7%)	11 (35.48%)/20 (64.5%)
Vascular invasion (Yes/No)	8 (50.0%)/8 (50.0%)	5 (33.3%)/10 (66.7%)	13 (41.9%)/18 (58.1%)
Extrahepatic metastases (Yes/No)	6 (37.5%)/10 (62.5%)	6 (40.0%)/9 (60.0%)	12 (38.7%)/19 (61.3%)

Continuous variables presented as mean value ± SD; categorical variables presented as absolute frequency and percentage.

**Table 2:** Incidence of adverse events with a frequency ≥4% in the study population.

Adverse Event	Total, *n* (%)	Control Group *n* (%)	Experimental Group *n* (%)
Total AE incidence	182	84 (46.2%)	98 (53.8%)
Total SAE incidence	19 (10.4%)	12 (63.2%)	7 (36.8%)
Gastrointestinal disorders	66 (36.3%)	39 (59.1%)	27 (40.9%)
Diarrhea	20	11 (55.0%)	9 (45.0%)
Abdominal pain	14	6 (42.9%)	8 (57.1%)
Anorexia/Hyporexia	9	3 (33.3%)	6 (66.7%)
Ascitis	8	6 (75.0%)	2 (25.0%)
Gastrointestinal hemorrhage	7	7 (100.0%)	0 (0.0%)
General disorders	33 (18.1%)	15 (45.4%)	18 (54.6%)
Asthenia	20	11 (55.0%)	9 (45.0%)
Weight loss	8	2 (25.0%)	6 (75.0%)
Skin and subcutaneous tissue disorders	30 (16.5%)	11 (36.7%)	19 (63.3%)
Hand-foot syndrome	9	5 (55.6%)	4 (44.4%)
Rash	8	1 (12.5%)	7 (87.5%)

## Supplementary Materials

The following are available online at https://www.mdpi.com/2072-6694/12/7/1900/s1, Table S1: Summary of survival results for the main variables.
