# Supplementary material for: Efficacy and Safety of the Combination of Pravastatin and Sorafenib for the Treatment of Advanced Hepatocellular Carcinoma (ESTAHEP Clinical Trial)
Source: Cancers (Basel). 2020 Jul 14;12(7):1900. doi: 10.3390/cancers12071900 (PMC7409102; doi:10.3390/cancers12071900)
Supplement: Supplementary file 1 [file cancers-12-01900-s001.pdf]

cancers

MDPI

Supplementary Materials: Efficacy and Safety of the Combination of Pravastatin and Sorafenib for the Treatment of Advanced Hepatocellular Carcinoma (ESTAHEP Clinical Trial)

Ioana Riaño, Leticia Martín, Maria Varela, Trinidad Serrano, Oscar Núñez, Beatriz Mínguez, Pedro M. Rodrigues, Maria J. Perugorria, Jesus M. Banales and Juan I. Arenas

Table S1. Summary of survival results for the main variables.

Population	Variable	*n*	Mean Survival (Months)	Median Survival (Months)	*p* Value
Total population	Overall survival	31	11.5	12.4	na
	Time to progression	28	7.6	7.5	na
	Time to symptomatic progression	31	6.2	4.6	na
	Treatment days: <129 vs. ≥129 days	15; 16	8.2 vs. 14.6	6.3 vs. 14.8	0.003
	Child-Pugh stage: A vs. B	28; 3	12.3 vs. 4.4	14.0 vs. 6.0	0.007
	Vascular invasion: YES vs. NO	13; 18	8.6 vs. 13.6	6.3 vs. 14.8	0.041
	Portal vein thrombosis: YES vs. NO	11; 20	8.5 vs. 13.1	6.3 vs. 14.8	0.026
	Etiology HCV vs. HBV vs. Alcohol	16; 3; 16	11.0 vs. 8.0 vs. 12.0	11.6 vs. 8.2 vs. 14.0	nd
	Tumoral response: PR vs. SD vs. PD	2; 11; 15	18 vs. 9.2 vs. 13.8	18 vs. 6.3 vs. 14.8	0.009
	Dermatological toxicity: YES vs. NO	16; 15	13.6 vs. 9.3	14.5 vs. 6.9	0.049
Treatment groups: sorafenib+placebo vs. sorafenib+pravastatin	Overall survival: sor+plac vs. sor+prav	16; 15	11.4 vs. 11.6	11.6 vs. 12.4	0.922
	Survival at the end of treatment: sor+plac vs. sor+prav	16; 15	6.1 vs. 8.6	3.2 vs. 9.7	0.188
	TTP: sor+plac vs. sor+prav	16; 15	4.3 vs. 9.8	3.2 vs. 9.9	0.008
	TTSP: sor+plac vs. sor+prav	16; 15	5.1 vs. 7.4	3.7 vs. 4.6	0.393
	Portal vein thrombosis YES: sor+plac vs. sor+prav	6;5	6.75 vs. 10.6	6.0 vs. 8.2	0.301
	Dose reduction NO: sor+plac vs. sor+prav	11; 10	10.9 vs. 12.1	11.6 vs. 12.4	0.705

HCV: hepatitis C virus; HBV: hepatitis B virus; PR, partial response; SD, stable disease; PD, progressive disease; sor, sorafenib; prav, pravastatin; TTP, time to progression; TTSP, time to symptomatic progression; na, not applicable; nd, not determined.

© 2020 by the authors. Submitted for possible open access publication under the terms and conditions of the Creative Commons Attribution (CC BY) license (http://creativecommons.org/licenses/by/4.0/).
